# Ovarian matrix metalloproteinases are differentially regulated during the estrous cycle but not during short photoperiod induced regression in Siberian hamsters (*Phodopus sungorus*)

**DOI:** 10.1186/1477-7827-8-79

**Published:** 2010-06-25

**Authors:** Lisa A Vrooman, Kelly A Young

**Affiliations:** 1 Reproductive Biology Group, Department of Biological Sciences, California State University, Long Beach, Long Beach, CA 90840, USA; 2 Current Address: Center for Reproductive Biology, PO Box 647521, Washington State University, Pullman, WA 99164-7521, USA

## Abstract

**Background:**

Matrix metalloproteinases (MMPs) are implicated as mediators for ovarian remodeling events, and are involved with ovarian recrudescence during seasonal breeding cycles in Siberian hamsters. However, involvement of these proteases as the photoinhibited ovary undergoes atrophy and regression had not been assessed. We hypothesized that 1) MMPs and their tissue inhibitors, the TIMPs would be present and differentially regulated during the normal estrous cycle in Siberian hamsters, and that 2) MMP/TIMP mRNA and protein levels would increase as inhibitory photoperiod induced ovarian degeneration.

**Methods:**

MMP-2, -9, -14 and TIMP-1 and -2 mRNA and protein were examined in the stages of estrous (proestrus [P], estrus [E], diestrus I [DI], and diestrus II [DII]) in Siberian hamsters, as well as after exposure to 3, 6, 9, and 12 weeks of inhibitory short photoperiod (SD).

**Results:**

MMP-9 exhibited a 1.6-1.8 fold decrease in mRNA expression in DII (p < 0.05), while all other MMPs and TIMPs tested showed no significant difference in mRNA expression in the estrous cycle. Extent of immunostaining for MMP-2 and -9 peaked in P and E then significantly declined in DI and DII (p < 0.05). Extent of immunostaining for MMP-14, TIMP-1, and TIMP-2 was significantly more abundant in P, E, and DI than in DII (p < 0.05). Localization of the MMPs and TIMPs had subtle differences, but immunostaining was predominant in granulosa and theca cells, with significant differences noted in staining intensity between preantral follicles, antral follicles, corpora lutea, and stroma classifications. No significant changes were observed in MMP and TIMP mRNA or extent of protein immunostaining with exposure to 3, 6, 9, or 12 weeks of SD, however protein was present and was localized to follicular and luteal steroidogenic cells.

**Conclusions:**

Although MMPs appear to be involved in the normal ovarian estrus cycle at the protein level in hamsters, those examined in the present study are unlikely to be key players in the slow atrophy of tissue as seen in Siberian hamster ovarian regression.

## Background

Normal ovarian function is dependent on a series of tissue remodeling events taking place throughout the reproductive cycle. For a number of species, including Siberian hamsters (*Phodopus sungorus*), a seasonal pattern of reproduction is exhibited in response to changes in photoperiod. Long (> 12 h of light per day) photoperiods correlate with abundant environmental resources for many temporal rodents, and therefore can stimulate reproductive physiology and behavior [1]. Follicle development, ovulation, and corpus luteum formation and degradation all occur during the four-day estrous cycle in Siberian hamsters with long day stimulation. In contrast, exposure to short (< 12 h of light per day) photoperiod can terminate reproductive function [1,2], inducing an anestrous/anovulatory state in regressed ovaries [3]. In Siberian hamsters, exposure to 12-14 weeks of short photoperiod results in reduced or absent ovulation and significant reductions in ovarian mass, the number of antral follicles and the number of corpora lutea (CL) [4-6].

In mammalian ovaries, the extracellular matrix (ECM) regulates cellular processes vital for follicle growth and maturation, including proliferation, differentiation, and survival [7], and its synthesis and degradation are vital to ovulation, CL formation, and luteal regression [8]. The remodeling of the ECM is mediated in part by a family of Zn^+^-dependent endopeptidases, matrix metalloproteinases (MMPs), and their tissue inhibitors (TIMPs). Signaled by a variety of hormones, growth factors, and cytokines, MMPs and TIMPs contribute to the degradation of the ECM in the ovary by cleaving the various tissue components to clear space for new growth [8,9]. MMP and TIMP protein levels and mRNA expression show distinct differences in expression during follicle development and ovulation, and throughout luteal formation and degradation in rats, mice, pigs, cattle, sheep, and primates, suggesting that the concerted action of MMPs may regulate these ovarian events [10-16].

The MMPs specifically investigated in this study are MMPs -2, -9, and -14 and TIMPs -1 and -2. MMP-2 and MMP-9, of the gelatinase class, promote follicle growth in both rodents and goats [11,17,18], and have been implicated in the ovulatory process. MMP-2 protein is localized to the granulosa and theca cells and both protein and mRNA are increased in rats following PMSG [10] or hCG administration [19], while in mice, MMP-9 mRNA expression is increased with LH stimulation [19]. Both MMP-2 and MMP-9 mRNA expressions increase in primate granulosa cells after hCG administration [20], and MMP-2 increases as the ovary returns to function in photostimulated Siberian hamsters [6]. MMP-14 (mt-MMP-1) is a transmembrane collagenase that cleaves collagens I, II, and III, as well as activates MMP-2 [21]. With the gonadotropin surge, MMP-14 mRNA is upregulated in bovine peri-ovulatory and luteal tissue [22], and increases in theca cells after ovulation is induced in mice [23]. Active MMP-14 protein is also increased in the bovine mid and late luteal periods [24], and MMP-14 mRNA is increased during photostimulated ovarian recrudescence in Siberian hamsters [6].

Both TIMP-1 and -2 are capable of inhibiting all MMP active forms, although show differential preference for certain MMPs. TIMP-1 preferentially inhibits MMP-9 [25], but is a poor inhibitor of MMP-14, and TIMP-2 forms a complex with pro-MMP-2 which can be stimulatory in conjunction with membrane bound MMPs, but effectively inhibits MMP-2 in higher concentrations [26]. TIMP-1 mRNA expression is increased after LH stimulus in rats [27], mice [23], and sheep [28], and TIMP-1 protein is localized to granulosa cells and luteal tissue following LH stimulus in sheep follicles [29]. In primates, TIMP-1 mRNA and protein and TIMP-2 mRNA are upregulated in human perifollicular ovarian stroma prior to and during ovulation [30] and TIMP-1 and -2 are upregulated in rhesus macaque periovulatory ovaries following hCG administration [20]. Finally, TIMP-1 declines during exposure to inhibitory photoperiods in Siberian hamsters and remains low throughout the bulk of recrudescence, returning to control levels only once the ovary returns to function [6].

While MMPs and TIMPs are linked to important events during the ovarian cycle across a number of species, and are dynamically expressed during photostimulated recrudescence in Siberian hamsters, the expression patterns of these MMPs/TIMPs during the hamster estrous cycle and during loss of function in photoperiod-induced ovarian regression have not been examined. In the current study, we hypothesized that MMP and TIMP mRNA and protein expression was 1) present and differentially regulated in the stages of estrous during tissue remodeling in the hamster estrous cycle, and 2) that MMPs and TIMPs are involved in the ovarian regression and loss of function. As a first step to address these hypotheses, mRNA and protein expression was determined in a select group of representative MMPs/TIMPs (MMP-2, -9, and -14 and TIMP-1 and -2) during the estrous cycle of Siberian hamsters subjected to a long day photoperiod (LD; 16L:8D) as well as in hamsters subjected to 3, 6, 9, and 12 weeks of short day photoperiod (SD; 8L:16D).

## Methods

### Animals

Adult Siberian hamsters (*Phodopus sungorus*) were purchased from the colony of Dr. Katherine Wynne-Edwards, Queens University (Kingston, Ontario, Canada). All procedures were performed at California State University, Long Beach and complied with the CSULB and National Research Council guidelines for use of laboratory animals. Animals were housed at 20 ± 2 °C in individual polypropylene cages equipped with bedding. Access to food (mixture of Lab Rodent Diet 5001 and Mazuri Hamster & Gerbil Diet, Purina, Brentwood, MO) and tap water was provided *ad libitum *for the duration of the experiment. All animals were acclimated to long day (LD) photoperiod conditions (16 h light: 8 h dark) for at least two weeks. Male hamsters were placed among females to maintain ovarian cyclicity, and estrous cycles were synchronized by placing soiled male bedding into the female cages four days prior to tissue collection [5].

At time of collection, body mass was measured, and the stage of the estrous cycle was initially determined by vaginal cytology. A cotton swab dampened with saline solution was inserted into the vagina and vaginal cells were smeared over a microscope slide for cytology. Once stage of estrous was estimated, animals were euthanized for tissue collection via cervical dislocation following a cocktail of ketamine (200 mg/kg) and xylazine (20 mg/kg). Ovaries were weighed, and one ovary was fixed in 10% neutral buffered formalin for 7 days, then transferred to 70% ethanol prior to paraffin embedding to confirm ovarian staging. The contralateral ovary was individually flash frozen for mRNA extraction. Blood samples were collected from the retro-orbital sinus, and plasma was stored at -80°C until radioimmunoassay to determine estradiol concentrations.

### Estrous cycle

Female hamsters were arbitrarily selected into the experimental groups of proestrus (P) (n = 7), estrus (E) (n = 6), diestrus I (DI) (n = 6), and diestrus II (DII) (n = 6). All tissue was collected between 0800 and 1000 h and within four days once each hamster was determined to be in the appropriate estrous cycle stage. An additional group of female hamsters (n = 16; 4 per estrous group) were collected similarly on a separate occasion for additional tissue for mRNA extraction.

### Reproductive regression

Tissue from female hamsters used for the regression portion of this study was taken from a complementary study in our laboratory [5]. These hamsters were housed in conditions described above, and subjected to 3, 6, 9, or 12 weeks of short (SD; 8 h light:16 h dark) or LD photoperiod after being acclimated to a long day photoperiod (n = 5-7 per group). Tissue was collected when vaginal smears indicated that females were in the DII portion of the estrous cycle, as this is the phase most closely resembling reproductive regression. Twelve weeks in SD results in reproductively inactive females with fully regressed ovaries lacking in antral follicles, ovulation, and estradiol production and containing terminal atretic follicles characteristic of regressed Siberian hamster ovaries [5].

### Estrous cycle: follicle counts

Formalin fixed tissues were dehydrated in a graded series of ethanol solutions and xylenes, and embedded in paraffin wax. Serial paraffin sections of 6 m thickness were collected from every 60 μm of tissue and mounted onto Superfrost-plus microscope slides (Fisher Scientific, Pittsburgh, PA). Tissues were stained with hematoxylin and eosin to differentiate ovarian structures. Ovarian structures were then counted according to the following groups: preantral follicles (one or more layers of cuboidal granulosa cells, no antrum present), antral follicles (multiple layers of granulosa cells, antrum present), atretic follicles (presence of >10 pyknotic granulosa nuclei and/or degrading oocyte), and corpora lutea. The average number of ovarian structures was determined from six sections per ovary per animal.

### Estrous cycle: radioimmunoassay

To confirm vaginal cytology and to correlate plasma concentrations of sex steroid hormones with MMP expression, estradiol concentrations were assessed. Following blood collection, plasma was subsequently separated by centrifugation (5000 rpm for 5 min) and stored at -80 °C until radioimmunoassay. Plasma estradiol concentrations were determined using the Ultra-Sensitive Estradiol RIA^125I ^double antibody kit (Diagnostic Systems Laboratories, Inc., Webster, TX). All samples were assayed in duplicate and their radioactivity was measured using a Perkin-Elmer Cobra II gamma counter (Packard Instruments Co., Boston MA). Values were entered into Sigma Plot software (SPSS Inc., Chicago, IL) and a standard curve was generated using the four-parameter logistic curve function. The final hormone concentrations were calculated using the Sigma Plot standard curve analysis function. Assay standards and controls were within the normal limits. Estradiol concentration values were compared against those of the CSULB Endocrine Laboratory and previous assays in Siberian hamsters [5,6]. The lower limits of detection for the estradiol assay was 5 pg/mL, with low cross-reactions to other steroids: 0.64-2.40%.

### RT-PCR/mRNA analysis

Following tissue collection, total RNA was isolated from the frozen ovaries using PureLink Micro-to-Midi Total RNA Purification System (Invitrogen, Carlsbad, CA) according to Invitrogen standard protocol. cDNA was generated by performing reverse transcription on all samples containing sufficient RNA using iScript cDNA Synthesis Kit (Bio-Rad Laboratories, Hercules, CA). GoTaq Green Promega PCR reagents (Promega, Madison, WI) were used to conduct semi-quantitative RT-PCR following previously optimized protocols with MMP primers [6].

For each female, 10 μl of PCR reaction was electrophoresed on a 2% agarose gel containing 1 μl of ethidium bromide to allow visualization. Gels were visualized using Bio-Rad Gel Doc SR documentation system (Bio-Rad, Hercules, CA), and the global adjusted volume of bands was analyzed using Quantity One software (The Discovery Series, Bio-Rad). The global adjusted volume of each gene was normalized by division of the global adjusted volume for the loading standard β-actin band to obtain relative mRNA expression.

### Immunohistochemistry

Sectioned ovary tissue was deparaffinized in xylene, rehydrated through a graded series of ethanol solutions, and washed in phosphate buffered saline (PBS). Antigen retrieval was then performed using Citra Antigen Unmasking Solution (Vector Laboratories, Burlingame, CA) in a pressure cooker for 10 min. Tissue was washed in PBS, then placed in 3% hydrogen peroxide/methanol solution for 10 minutes and blocked using the appropriate 10% serum (Vector Laboratories, Burlingame, CA). Horse serum was used for the monoclonal MMPs -2, -14, and TIMP-2, and goat serum was used for the polyclonal MMP-9 and TIMP-1, then incubated for 45 minutes at room temperature. The primary antibodies for MMP-2 and -9 (pro and active forms), MMP-14, TIMP-1, and TIMP-2 (active forms) (Chemicon International, Temecula, CA) were applied at the appropriate dilution (MMP-2, -9 and TIMP-2 at 1:200, TIMP-1 at 1:400, and MMP-14 at 1:800) to tissue, incubated for 1 hour at room temperature, then overnight at 4 °C. Tissue was washed with PBS and incubated for 45 minutes with anti-mouse (MMP-2, MMP-14, and TIMP-2) or anti-rabbit (MMP-9 and TIMP-1) antibody prior to incubation for 30 min in an avidin-biotin peroxidase solution (Vectastain Elite ABC kit; Vector Laboratories, Burlingame, CA). Vector NovaRED Substrate Kit (Vector Laboratories) was used to detect the protein expression followed by counterstaining with hematoxylin.

Intensity of immunostaining was noted for each MMP or TIMP for preantral follicles, antral follicles, corpora lutea, terminal atretic follicles (characteristic of regressed ovarian tissue in Siberian hamsters), stroma between follicles/CL comprised primarily of connective/endothelial tissue, and stroma containing potentially steroidogenic interstitial cells not incorporated into a defined follicle [31]. Structures in each section were given a numerical value ranging from 0-4. A score of 0 indicated no staining; a score of 1 meant some faint staining in the structures of the subtype being scored, a score of 2 indicated light staining in the structures of the subtype being scored, a score of 3 specified medium-intense staining in the structures of the subtype being scored, and a score of 4 specified intense staining in the structures of the subtype being scored. A minimum of three animals showing each structure type was required for analysis of that structure type, therefore CL tissue was not scored in P, terminal atretic follicles were not scored in LD and SD week 3 animals, and antral follicles and CL were not scored in regressed tissue. Additionally, extent of staining across the ovary was noted for three cross sections per animal per MMP or TIMP, and assessed using an immunostaining index. Sections were given a numerical value ranging from 0-4. A score of 0 indicated no staining; a score of 1 meant staining across ~25% of the structures/stroma in the cross section, a score of 2 indicated staining across ~50% of the structures/stroma of the cross section, a score of 3 specified staining across ~75% of the structures/stroma of the cross section, and a score of 4 specified intense staining ~100 of structures/stroma in the cross section. For all counts, scores for three sections (60-100 microns apart at minimum) per animal were averaged, and included in the group mean (n = 5-7 animals per group) used in the ANOVA analysis.

### Statistical analysis

All data were analyzed using Prism 4 statistical software 240 package (GraphPad Software, Inc. San Diego, CA). One-way ANOVAs were performed on all groups and represented by mean ± SEM. If results were significant (p < 0.05) with a 95% confidence interval, a Neuman-Keuls post-hoc test was used to compare experimental groups. A logY transformation was used to determine statistical differences in MMP and TIMP estrous cycle mRNA data to reduce variance.

## Results

### Estrous cycle: follicular analysis

To confirm estrous cycle stage, follicle counts were quantified for the number of preantral, antral, and atretic follicles as well as corpora lutea. The number of preantral follicles was 2.5-fold lower in DII compared to P and 2.2-fold lower compared to E (p < 0.01). The number of antral follicles significantly increased 2.8-fold in E compared to P (p < 0.01) and 1.8-fold as compared to DI (p < 0.05), and were nearly absent in DII altogether (p < 0.001). However, atretic follicles (not categorized by size or stage of atresia) were 3- 4.5-fold more abundant in DII in comparison to all other estrous stages (p < 0.01). The number of CL present increased nearly 10-fold from P to DII (p < 0.01; Table 1).

**Table 1:** Follicles and corpora lutea per ovarian section in LD Siberian hamster estrous cycle

Estrous group	Preantral	Antral	Atretic	Corpora Lutea
**Proestrus**	7.00 (0.7)^a^	0.72 (0.2)^ac^	0.77 (0.2)^a^	0.10 (0.0)^a^
**Estrus**	6.17 (0.8)^a^	2.02 (0.5)^b^	1.13 (0.5)^a^	0.24 (0.1)^a^
**Diestrus I**	4.77 (1.0)^ab^	1.10 (0.2)^a^	0.97 (0.1)^a^	0.90 (0.2)^b^
**Diestrus II**	2.79 (0.5)^b^	0.15 (0.1)^c^	3.43 (0.7)^b^	0.97 (0.1)^b^

Values represented as mean (SEM). Groups with different letters are significantly different (p < 0.05).

### Estrous cycle: radioimmunoassay

**Figure 1:**
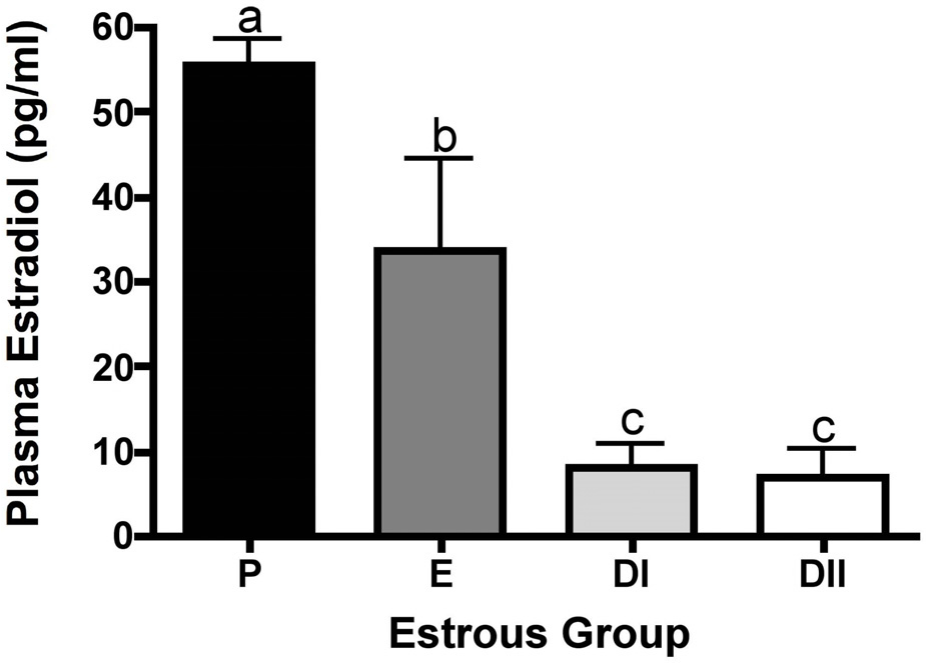
**Plasma estradiol concentrations (pg/ml) in the Siberian hamster estrous cycle**. Bar graphs represent mean ± SEM. Groups with different letters are significantly different (p < 0.05).

Plasma estradiol concentrations were measured by radioimmunoassay. Estradiol concentration peaked in P, declining 1.7-fold in E (p < 0.05) and 6.9-fold by DI (p < 0.01), to a final 8.1-fold decrease by DII (p < 0.001; Figure 1).

### Estrous cycle: RT-PCR/mRNA analysis

**Figure 2:**
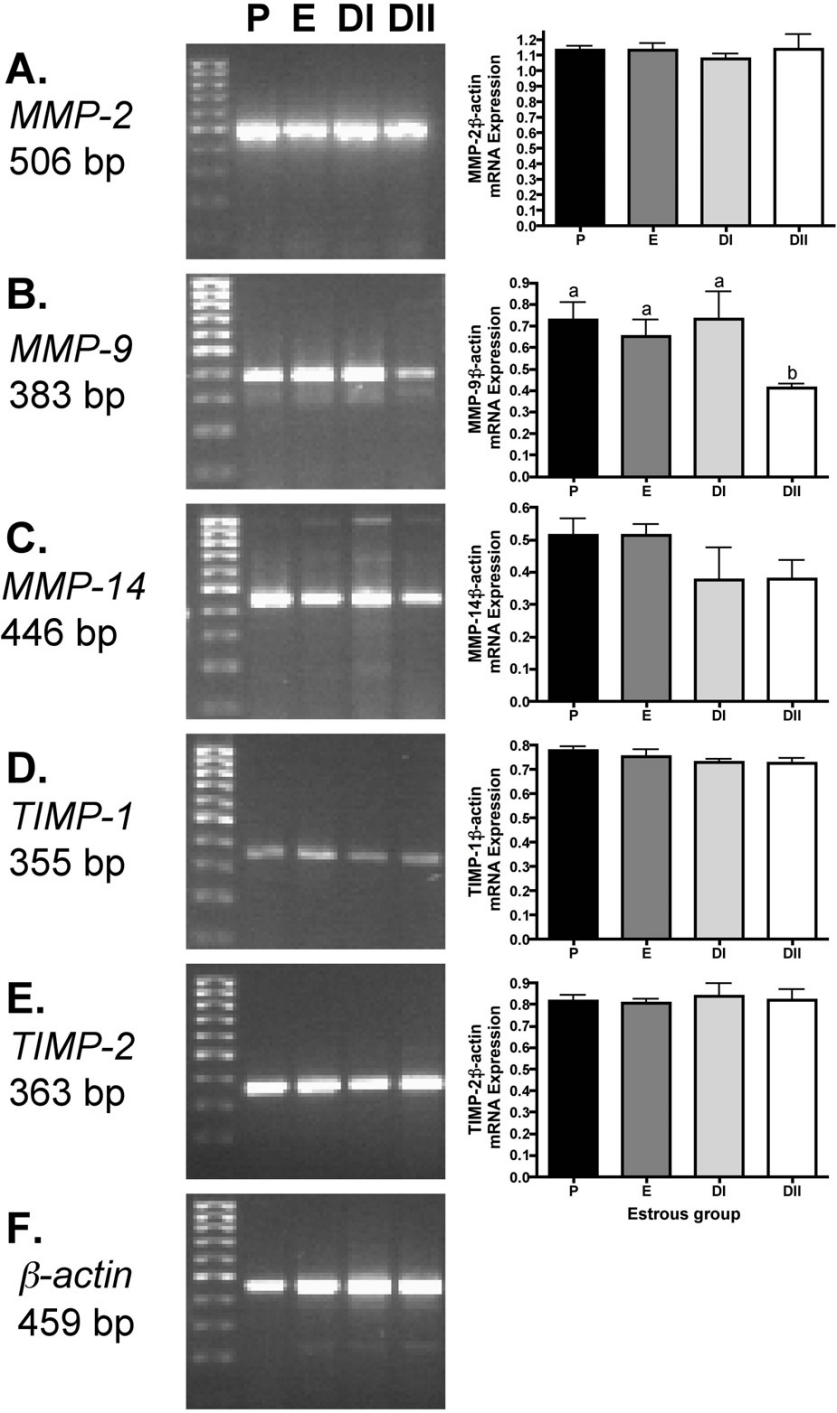
**Semi-quantitative RT-PCR expression of MMP and TIMP mRNA in the Siberian hamster estrous cycle**. (A) MMP-2, (B) MMP-9, (C) MMP-14, (D) TIMP-1, (E) TIMP-2, and (F) β-actin used as a control gene for all RT-PCRs. Bar graphs represent mean ± SEM relative levels mRNA expression of MMP and TIMPs:β-actin mRNA expression. Groups with different letters are significantly different (p < 0.05)

Total ovarian mRNA for MMPs-2, -9, -14 and TIMP-1 and TIMP-2 was analyzed by RT-PCR to determine differences in relative mRNA expression between estrous groups. MMP -2 (Figure 2A), MMP-14 (Figure 2C) as well as TIMP-1 (Figure 2D) and TIMP-2 (Figure 2E) showed no significant differences between the stages of estrous. However, MMP-9 displayed a 1.6-1.8 decline in relative mRNA expression in DII in comparison to the other groups (p < 0.05; Figure 2B). Beta actin mRNA expression was used to normalize MMP/TIMP expression and showed no changes (Figure 2F).

### Estrous cycle: immunohistochemistry

**Figure 3:**
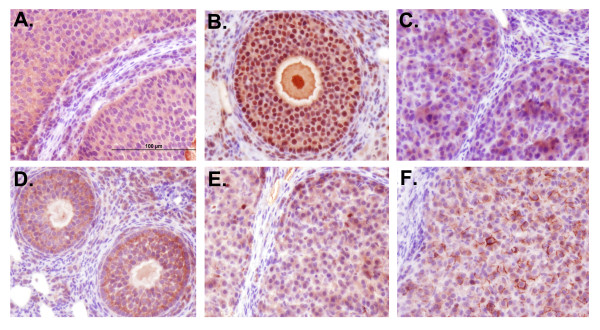
**Representative Detail of MMP and TIMP protein immunodetection in Siberian hamsters**. Ovarian immunohistochemical staining (dark red stain on purple/blue hematoxylin background). (A) MMP-2 immunostaining, diestrus I detail, (B) MMP-9 immunostaining, diestrus I detail, (C) MMP-14 immunostaining, estrus detail, (D) TIMP-1 immunostaining, estrus detail, (E) TIMP-2 immunostaining, estrus detail showing mostly cytoplasmic staining, and (F) TIMP-2 immunostaining, proestrus detail showing cytoplasmic and membrane immunostaining.

**Figure 4:**
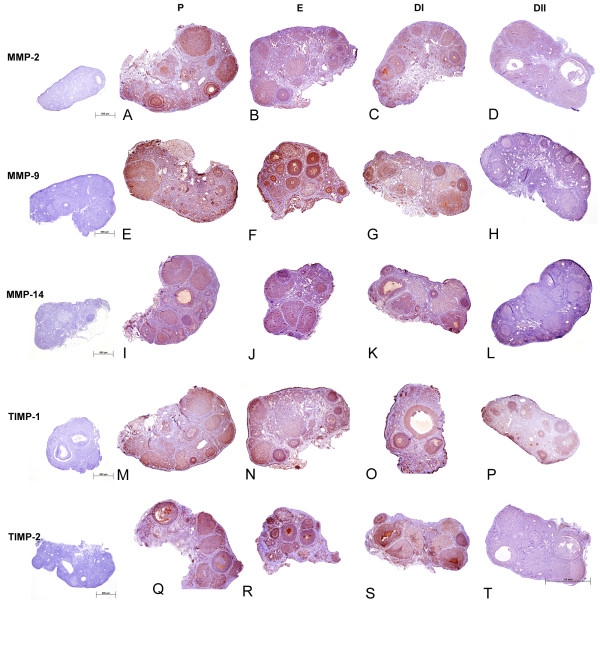
**Representative MMP and TIMP protein immunodetection during the estrous cycle in Siberian hamsters**. Ovarian immunohistochemical staining (dark red stain on purple/blue hematoxylin background) for all MMPs and TIMPs. (A-D) MMP-2 immunostaining, (E-H) MMP-9 immunostaining, (I-L) MMP-14 immunostaining, (M-P) TIMP-1 immunostaining, and (Q-T) TIMP-2 immunostaining. Insets show negative control immunostaining (no primary antibody present).

Primary anti-mouse antibodies for MMPs-2, 9, and 14 and TIMPs-1 and -2 were used on paraffin embedded tissue sections (Figures 3 and 4). No staining was observed in control sections processed without primary antibodies (Figure 4, insets). To confirm specificity in hamster tissue, mouse ovaries were also used, and staining patterns in these positive controls matched what was observed in the hamster ovaries for all antibodies (data not shown). All MMP and TIMP protein expression displayed cytoplasmic staining present at some level in all stages of estrous; staining was diffuse in MMP-2 (Figure 3A), MMP-14 (Figure 3C), and the TIMPs (Figure 3D,E), and tended to concentrate around the nucleus for MMP-9 (Figure 3B). In addition to cytoplasmic immunostaining, membrane staining was also observed in TIMP-2 stained sections (Figure 3F).

MMP-2 immunostaining showed abundant localization in granulosa, theca, and scattered stromal cells in P (Figure 4A) and E (Figure 4B) and DI (Figure 4C). In DII immunoreactivity was minimal and localized predominantly to steroidogenic cells (Figure 4D). MMP-9 protein was present in all stages of the estrous cycle, exhibiting cytoplasmic staining that tended to concentrate around the nucleus (Figure 3B). Staining was localized to granulosa, theca, and scattered stroma in P, E, and DI, with some CL staining more intensely than others (Figures 4E-EG). In DII, immunostaining was minimal, and was localized primarily to preantral granulosa cells with occasional staining observed in CL and atretic follicles (Figure 4H). Both MMP-2 and MMP-9 displayed intense basal membrane staining in P and E follicles. MMP-14 immunostaining was localized to granulosa, theca, and scattered stroma in P and E (Figures 4I and 4J). In DI, the same level of staining was observed, however a differentiation of staining between preantral and antral follicles was observed; smaller follicles displaying slightly higher staining and larger antral follicles displaying slightly lower staining (Figure 4K). Different levels of staining among CLs were also exhibited in P, E, and DI. In DII, staining was localized to the granulosa of select preantral follicles and CL (Figure 4L).

Immunostaining for TIMP-1 was localized to granulosa, theca, and scattered stromal cells, in addition to the basal membrane in P and E follicles (Figure 4M and 4N). By DII, staining was localized only to the granulosa cells of follicles and CL (Figure 4P). TIMP-2 staining was localized to steroidogenic cells with minimal stroma staining (Figure 4Q-S). Staining was more intense in the granulosa and theca of larger antral follicles, than in the smaller antral follicles and preantral follicles. Staining in diestrus I was localized to steroidogenic and some stromal cells (Figure 4S), with staining observed only in granulosa cells in DII (Figure 4T).

Both intensity and extent of immunostaining was quantified. Intensity was scored for individual follicular structures (Table 2). MMP-2 immunostaining intensity in preantral follicles declined 4.5- 5.7 fold in DII as compared to P, E, and DI, whereas MMP-2 immunoreactivity was lowest in DI for antral follicles (p < 0.05; Table 2). MMP-2 immunostaining was also low in DII for CL tissue as compared to E and DI, and in potentially steroidogenic stroma, in DII as compared to all other groups (p < 0.05, Table 2). Staining in stroma consisting primarily of connective tissue (CT) peaked in P, as compared to other groups; however overall intensity was still low (p < 0.05; Table 2). Intensity of MMP-9 immunostaining for preantral follicles, antral follicles, CL, and potentially steroidogenic stroma was reduced 2.7- 4.3 fold in DII as compared to all other groups, while no changes were observed in the low intensity observed in stroma consisting primarily of CT (Table 2). MMP-14 intensity was also reduced 2.5- 4.6 fold in preantral follicles, antral follicles, CL and potentially steroidogenic stroma in DII tissue as compared to all other groups; whereas immunostaining intensity was lowest in both DI and DII for connective tissue based stroma, dropping 3.9- 10.7 fold (p < 0.05; Table 2). TIMP-1 immunostaining intensity declined 1.2- 1.3 fold in DI as compared to P and E stages, then decreased 1.6- 1.7 fold in DII as compared to P and E (p < 0.05; Table 2). Antral follicle TIMP-1 immunostaining intensity declined 1.4- 1.6-fold in DI and DII as compared to P and E (p < 0.05); however, no changes were observed in intensity of immunostaining in CL or in the low levels of staining observed in connective tissue-based stroma (p > 0.5; Table 2). In contrast, TIMP-1 immunoreactivity in stroma containing potentially steroidogenic cells declined 16.0-19.4 fold in DI as compared to all other groups (p < 0.05). Finally, immunoreactivity for both preantral and antral follicles declined 11.2- 21.8 fold in DI as compared to all other groups (p, 0.05; Table 2). Immunostaining in CL tissues for TIMP-2 declined 1.1-fold between E and DI, and no staining was noted in CL in DII (p < 0.05; Table 2). Finally, no significant differences were noted in the low staining in connective tissue-based stroma (p > 0.05), whereas immunoreactivity peaked in potentially steroidogenic stroma in E, and declined 12.5-fold by DII (p < 0.05; Table 2).

**Table 2:** Immunostaining intensity index for MMPs and TIMPs in estrous cycle tissues

	Proestrus	Estrus	Diestrus I	Diestrus II
**MMP-2**				
Preantral	3.43 (0.8)^a^	3.08 (0.6) ^a^	2.67 (0.9) ^a^	0.60 (0.5) ^b^
Antral	3.51 (0.3) ^a^	2.96 (0.3) ^a^	0.22 (0.1) ^b^	2.93 (0.7) ^a^
CL	N/A	2.70 (0.3) ^a^	2.63 (0.4) ^a^	0.83 (0.2) ^b^
Stroma (CT)	0.65 (0.2) ^a^	0.00 (0.0) ^b^	0.00 (0.0) ^b^	0.00 (0.0) ^b^
Stroma (Steroidogenic)	2.24 (0.2) ^a^	2.25 (0.4) ^a^	2.52 (0.4) ^a^	0.83 (0.2) ^b^
**MMP-9**				
Preantral	3.83 (0.8) ^a^	3.96 (0.0) ^a^	3.86 (0.1) ^a^	1.67 (0.2) ^b^
Antral	3.57 (0.1) ^a^	3.83 (0.2) ^a^	3.93 (0.1) ^a^	1.33 (0.2) ^b^
CL	N/A	3.53 (0.2) ^a^	3.40 (0.3) ^a^	1.25 (0.3) ^b^
Stroma (CT)	0.42 (0.2)	0.17 (0.1)	0.27 (0.1)	0.00 (0.0)
Stroma (Steroidogenic)	2.70 (0.2) ^a^	3.37 (0.2) ^a^	2.93 (0.3) ^a^	0.78 (0.1) ^b^
**MMP-14**				
Preantral	2.93 (0.4) ^a^	2.63 (0.2) ^a^	3.40 (0.2) ^a^	0.74 (0.3) ^b^
Antral	2.45 (0.2) ^a^	2.56 (0.3) ^a^	2.99 (0.2) ^a^	0.83 (0.4) ^b^
CL	N/A	2.67 (0.1) ^a^	3.07 (0.2) ^a^	1.05 (0.3) ^b^
Stroma (CT)	2.14 (0.1) ^a^	2.07 (0.1) ^a^	0.20 (0.1) ^b^	0.53 (0.2) ^b^
Stroma (Steroidogenic)	2.14 (0.1) ^a^	2.10 (0.1) ^a^	2.43 (0.2) ^a^	0.78 (0.2)^b^
**TIMP-1**				
Preantral	3.44 (0.1) ^a^	3.22 (0.2) ^a,b^	2.633 (0.3)^b^	1.97 (0.3) ^c^
Antral	3.19 (0.1) ^a^	3.06 (0.3) ^a^	2.23 (0.1) ^b^	2.00 (0.0) ^b^
CL	N/A	2.55 (0.2)	1.98 (0.2)	1.42 (0.4)
Stroma (CT)	0.00 (0.0)	0.00 (0.0)	0.10 (0.1)	0.10 (0.1)
Stroma (Steroidogenic)	2.58 (0.1) ^a^	2.47 (0.2) ^a^	2.13 (0.2) ^a^	0.133 (0.2) ^b^
**TIMP-2**				
Preantral	3.39 (0.2) ^a^	3.40 (0.2) ^a^	2.80 (0.3) ^a^	0.25 (0.2) ^b^
Antral	3.39 (0.2) ^a^	3.70 (0.1) ^a^	3.60 (0.1) ^a^	0.17 (0.2) ^b^
CL	N/A	3.57 (0.1) ^a^	3.13 (0.1) ^b^	0.00 (0.0) ^c^
Stroma (CT)	0.00 (0.0)	0.07 (0.1)	0.00 (0.0)	0.00 (0.0)
Stroma (Steroidogenic)	3.03 (0.3) ^a^	3.87 (0.1) ^b^	3.05 (0.2) ^a^	0.31 (0.2) ^c^

Individual indices presented by ovarian structure type: preantral follicles, antral follicles, corpora lutea (CL), stroma primarily consisting of connective tissue (CT), stroma including potential steroidogenic interstitial glands. Values represented as mean (SEM). Groups with different letters are significantly different (p < 0.05).

**Figure 5:**
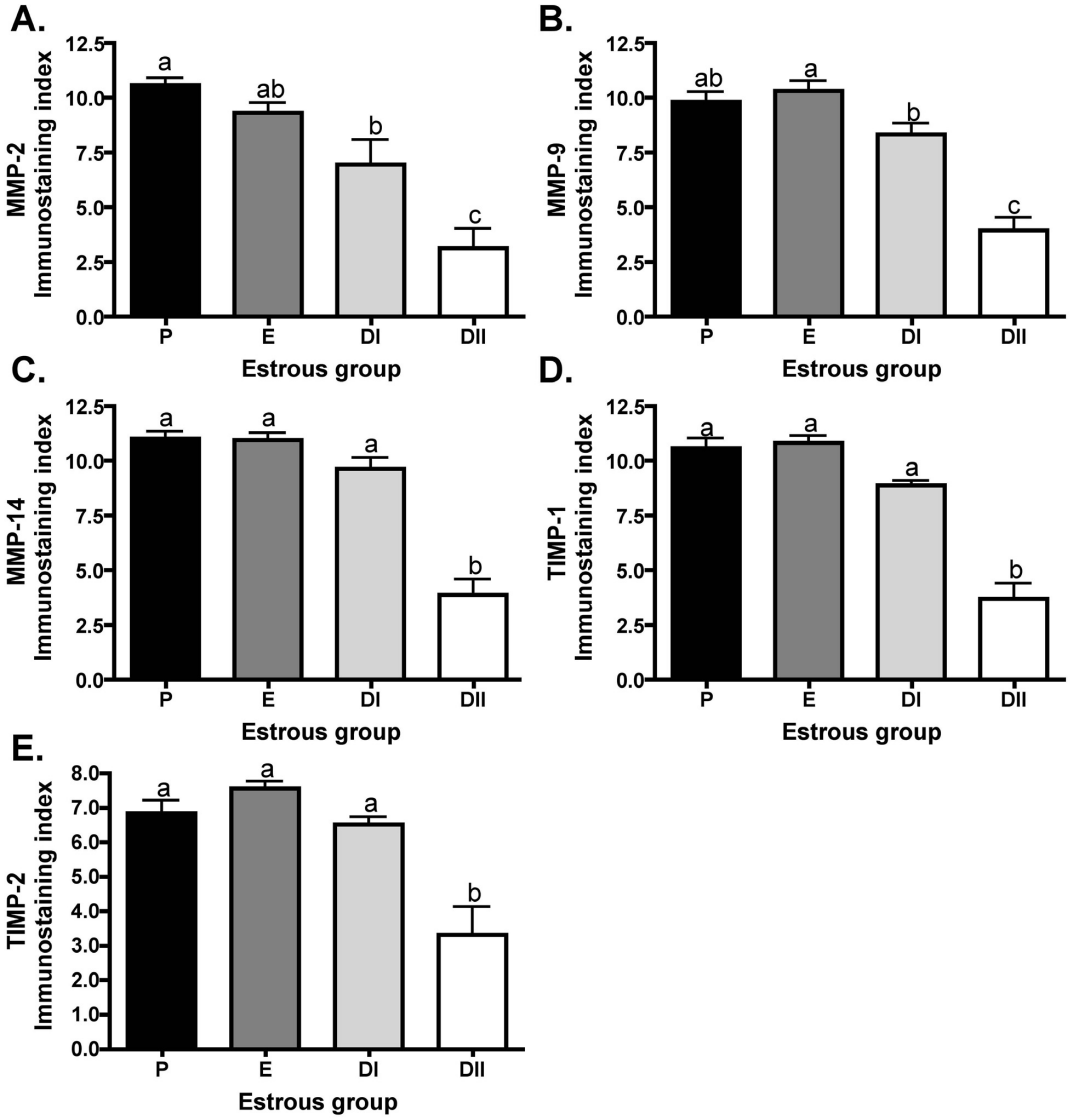
**Extent of immunostaining for MMPs and TIMPs during the estrous cycle**. Bar graphs represent mean ± SEM immunostaining index levels (scores of 0-4) for (A) MMP-2, (B) MMP-9, (C) MMP-14, (D) TIMP-1, and (E) TIMP-2. Index indicates overall extent dark red stained cells across the ovarian cross section in the different stages of estrous in Siberian hamsters. Groups with different letters are significantly different (p < 0.05).

The overall extent of protein expression was similar among all MMPs and TIMPs, exhibiting higher protein expression in P and E, and lower protein expression in DII (Figure 5). Total extent of ovarian immunostaining observed for MMP-2 peaked in P and E then declined in DI (p < 0.05) and again in DII (p < 0.01; Figure 5A). Extent of MMP-9 staining across the ovarian cross sections peaked in P and E, then declined 1.2-fold in DI (p < 0.01), and 3.4-fold in DII (p < 0.001; Figure 5B). Extent of MMP-14 staining was abundant in P, E, and DI then declined 3-fold in DII (p < 0.001; Figure 5C). Extent of immunostaining for TIMP-1 was significantly more abundant in P, E, and DI than in DII, declining 3-fold in DII (p < 0.001; Figure 5D). Extent of TIMP-2 staining across the ovarian cross sections peaked in P, E, and DI and declined in diestrus II (p < 0.01; Figure 5E).

### Regression: RT-PCR/mRNA analysis

Total ovarian mRNA for MMPs-2, -9, -14 and TIMP-1 and TIMP-2 were analyzed by RT-PCR to determine differences in mRNA expression with induced SD photoperiod. Hamsters in LD (DII stage) showed no difference from those in DII in the estrous cycle experiment. Exposure to 3, 6, 9, or 12 weeks of SD did not alter mRNA expression of any of the MMPs or TIMPs tested, as no significant differences among the groups were observed (p > 0.05; Table 3).

**Table 3:** Ovarian mRNA expression for MMPs and TIMPs during photoperiod induced regression

	Long Day	SD Week 3	SD Week 6	SD Week 9	SD 12
**MMP-2**	0.63 (0.0)	0.57 (0.1)	0.58 (0.1)	0.57 (0.1)	0.61 (0.1)
**MMP-9**	0.59 (0.0)	0.54 (0.0)	0.52 (0.0)	0.62 (0.0)	0.52 (0.1)
**MMP-14**	0.63 (0.1)	0.67 (0.0)	0.67 (0.1)	0.65 (0.1)	0.55 (0.1)
**TIMP-1**	0.72 (0.1)	0.83 (0.1)	0.76 (0.0)	0.73 (0.1)	0.71 (0.1)
**TIMP-2**	0.76 (0.0)	0.78 (0.1)	0.76 (0.0)	0.70 (0.1)	0.70 (0.0)

Relative values normalized to Beta Actin and represented as mean (SEM). No significant differences noted.

### Regression: immunohistochemistry

All MMPs and TIMPs examined were present at some level in all weeks of regression and displayed cytoplasmic staining. Staining was diffuse for MMPs-2, -14, and the TIMPs, and tended to concentrate around the nucleus for MMP-9. Staining patterns were similar to that observed in the estrous cycle (Figure 4), and are described here briefly, but not shown. MMP-2 staining was observed in the granulosa cells of most follicles and CL, with scattered stromal staining. Staining for MMP-9 was cytoplasmic, but tended to concentrate around the nucleus in steroidogenic cells, with noticeable staining in atretic follicles present in weeks 9 and 12. MMP-14 immunostaining was present in steroidogenic cells in all weeks, and was localized only to degenerating CLs and leutinizing atretic follicles (LAFs) in weeks 9 and 12. TIMP-1 staining was localized to the granulosa of all follicles, the theca of select follicles, and scattered stroma in all weeks. TIMP-2 showed consistent localization throughout SD weeks, showing minimal staining in all granulosa and patchy stromal staining.

**Figure 6:**
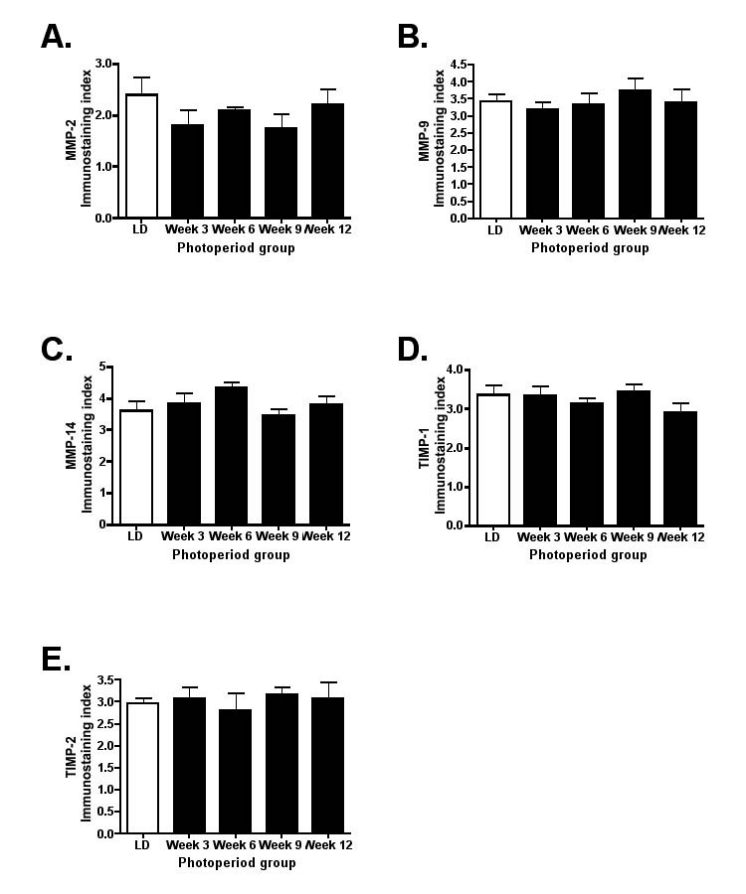
**Extent of immunostaining for MMPs and TIMPs during gonadal regression**. Bar graphs represent mean ± SEM immunostaining index levels (scores of 0-4) for (A) MMP-2, (B) MMP-9, (C) MMP-14, (D) TIMP-1, and (E) TIMP-2. Index indicates overall extent of dark red stained cells across the ovarian cross section in the different stages of estrous in Siberian hamsters. No differences were noted (p > 0.05).

Intensity and extent of staining was quantified using the immunostaining index. Intensity was scored for individual follicular structures (Table 4). MMP-2 immunostaining did not differ significantly across regression tissues when preantral follicles, antral follicles, CL, terminal atretic follicles typical of regressed tissue, and connective tissue stroma were assessed (p < 0.05); however, potentially steroidogenic stromal tissues showed an increase in staining intensity after 12 weeks in SD photoperiod (p < 0.05; Table 4). Immunostaining intensity for MMP-9 did not differ across groups for any structure (p > 0.05). Similarly, MMP-14 immunostaining intensity did not differ significantly across groups for preantral follicles, antral follicles, CL, or stroma types (p > 0.05); whereas terminal atretic follicles stained more intensely following 12 weeks of SD exposure as compared to 6 and 9 weeks in SD (p < 0.05; Table 4). TIMP-1 immunostaining intensity also peaked at 12 weeks of SD exposure as compared to week 6 and week 9 for terminal atretic follicles (p < 0.05), although immunoreactivity intensities for other structures did not differ across groups (p > 0.05; Table 4). Finally, TIMP-2 immunostaining intensity did not differ for any ovarian structure across groups (p > 0.05; Table 4). When overall extent of staining across the ovarian cross section was assessed, no differences were noted for any MMP or TIMP for any group (p < 0.05; Figure 6).

**Table 4:** Immunostaining intensity index for MMPs and TIMPs in regression tissues

	Long Day	Week 3	Week 6	Week 9	Week 12
**MMP-2**					
Preantral	1.19 (0.2)	1.27 (0.0)	1.02 (0.2)	0.69 (0.1)	1.31 (0.4)
Antral	1.03 (0.2)	0.92 (0.1)	N/A	N/A	N/A
CL	1.01 (0.1)	1.00 (0.1)	N/A	N/A	N/A
TAF	N/A	N/A	0.91 (0.2)	0.94 (0.1)	1.44 (0.3)
Stroma (CT)	0.02 (0.0)	0.08 (0.1)	0.06 (0.1)	0.00 (0.0)	0.00 (0.0)
Stroma (Steroidogenic)	1.68 (0.3) ^a^	1.08 (0.2) ^a^	1.63 (0.3) ^a^	0.89 (0.1) ^a^	2.94 (0.5) ^b^
**MMP-9**					
Preantral	1.40 (0.1)	1.28 (0.3)	1.04 (0.2)	1.07 (0.1)	1.24 (0.3)
Antral	1.23 (0.2)	1.28 (0.2)	N/A	N/A	N/A
CL	1.05 (0.9)	0.94 (0.6)	N/A	N/A	N/A
TAF	N/A	N/A	0.87 (0.2)	1.04 (0.4)	1.67 (0.2)
Stroma (CT)	0.01 (0.0)	0.00 (0.0)	0.00 (0.0)	0.00 (0.0)	0.00 (0.0)
Stroma (Steroidogenic)	1.08 (0.1)	1.19 (0.2)	1.17 (0.2)	1.07 (0.2)	1.57 (0.1)
**MMP-14**					
Preantral	0.87 (0.3)	0.48 (0.2)	0.27 (0.1)	0.08 (0.1)	0.34 (0.3)
Antral	0.67 (0.2)	0.60 (0.2)	N/A	N/A	N/A
CL	0.55 (0.2)	0.85 (0.3)	N/A	N/A	N/A
TAF	N/A	N/A	0.31 (0.1) ^a^	1.14 (0.2) ^a^	1.34 (0.3) ^b^
Stroma (CT)	0.06 (0.0)	0.05 (0.1)	0.00 (0.0)	0.00 (0.0)	0.00 (0.0)
Stroma (Steroidogenic)	0.76 (0.2)	0.57 (0.2)	0.40 (0.1)	0.11 (0.1)	0.42 (0.3)
**TIMP-1**					
Preantral	1.87 (0.2)	1.60 (0.2)	1.75 (0.2)	2.25 (0.1)	2.28 (0.2)
Antral	1.85 (0.1)	1.47 (0.2)	N/A	N/A	N/A
CL	1.43 (0.4)	1.35 (0.2)	N/A	N/A	N/A
TAF	N/A	N/A	1.19 (0.1) ^a^	2.31 (0.1)^b^	2.08 (0.4)^b^
Stroma (CT)	0.07 (0.1)	0.00 (0.0)	0.00 (0.0)	0.00 (0.0)	0.00 (0.0)
Stroma (Steroidogenic)	1.41 (0.2)	1.60 (0.2)	1.65 (0.2)	2.22 (0.1)	1.46 (0.2)
**TIMP-2**					
Preantral	0.85 (0.1)	0.85 (0.2)	0.97 (0.2)	0.98 (0.2)	1.23 (0.3)
Antral	0.83 (0.1)	0.71 (0.1)	N/A	N/A	N/A
CL (early)	0.83 (0.1)	0.83 (0.1)	N/A	N/A	N/A
TAF	N/A	N/A	0.87 (0.1)	0.81 (0.3)	1.07 (0.2)
Stroma (CT)	0.00 (0.0)	0.00 (0.0)	0.00 (0.0)	0.00 (0.0)	0.00 (0.0)
Stroma (Steroidogenic)	0.90 (0.1)	0.71 (0.1)	0.67 (0.1)	0.69 (0.3)	1.26 (0.3)

Individual indices presented by ovarian structure type: preantral follicles, antral follicles, corpora lutea (CL), terminal atretic follicles (TAF), stroma primarily consisting of connective tissue (CT), stroma including potential steroidogenic interstitial glands. Values represented as mean (SEM). Groups with different letters are significantly different (p < 0.05).

## Discussion

Ovaries of Siberian hamsters, like those of other seasonal breeders, can transition from fully functional to a quiescent, non-functional state and subsequently perform the reverse action, under the influence of photoperiod alone. This study examines for the first time MMP and TIMP involvement in Siberian hamster ovarian remodeling during the estrous cycle and photoperiod-induced ovarian regression. Because MMPs and TIMPs are differentially regulated during the photoperiod-mediated return of ovarian function [6], we hypothesized that MMP and TIMP mRNA and protein expression was present and differentially regulated in the hamster estrous cycle and that MMPs and TIMPs were involved in photoperiod induced ovarian regression. Our results add to the wealth of evidence supporting MMP and TIMP action in ovarian remodeling during the estrous cycle, especially in follicular growth and corpus luteum formation, as well as begin to elucidate MMP and TIMP roles in seasonal reproduction.

Follicular development and plasma estradiol concentrations were analyzed to contribute to our characterization of Siberian hamster ovaries throughout the estrous cycle. The number of antral follicles from our morning ovarian tissue collection peaked during early E, with the peak CL numbers occurring in the diestrus stages. These events follow the typical pattern of early E ovulation, followed by CL formation. In mice, large preovulatory follicles peak in P, and are reduced following ovulation in E when tissue is collected in late estrus, and large CL numbers peak in diestrus phases [32]. Atretic follicles (not categorized by size or stage of atresia) also peaked during DII (Table 1), similar to findings of increased larger atretic follicles during metaestrus (DI) and diestrus (DII) in rats and mice [32,33], and increased numbers of small atretic follicles in mouse metaestrus [32]. Results from the estradiol radioimmunoassay support the correct assignment to estrous group, as animals show the expected peak in plasma estradiol in P and gradual decline in latter groups, similar to the peak in P and decline in DI observed in previous studies of LD Siberian hamsters [34].

MMP and TIMP analysis during the estrous cycle indicated that both mRNA and protein for the MMPs and TIMPs examined were present in Siberian hamster ovaries and that MMP protein exhibited dynamic change across the cycle stages. In the present study, mRNA for MMPs -2 and -14 and TIMPs -1 and -2 failed to show a significant difference across the natural estrous cycle. Although many MMP expression studies use hyperstimulated and primed animals, and thus are difficult to compare, our data are consistent with MMP mRNA expression in unstimulated mice, which exhibit no changes in expression across the estrous phases [35]. In contrast, a significant peak in TIMP-1 mRNA during E and a metestrus (DI) and diestrus (DII) decline in TIMP-2 mRNA (3.5 kb transcript) have been reported for naturally cycling rats using Northern blot techniques, although the 1.0 kb TIMP-2 transcript showed no changes across the cycle [12]. Species and technique differences may partially explain these differences. Our data support that MMP proteins exhibit changes across the estrous cycle and may be regulated by endogenous hormones, but not to the extent as in primed animals that also exhibit changes at the mRNA level. Additionally, all MMPs are produced as zymogens, or pro-MMPs, which may explain why MMPs that differ with immunostaining levels express no change with mRNA. Post-translational regulation is an efficient way of fine-tuning MMP action, which would be especially crucial during the rapid changes occurring in the ovary through the hamster estrous cycle. Possible candidates for this upregulation are known MMP activators, extracellular matrix metalloproteinase inducer (EMMPRIN) [36] or the plasminogen activator-plasmin system [37,38]. It is also possible that other members of the MMP family that were not analyzed in our study show changes in mRNA expression. Since total ovarian MMP and TIMP mRNA was measured, this may not reflect the changes observed in specific cell types, especially granulosa and theca cells which comprise the growing structures in the ovary.

Extent of immunoreactivity for MMP and TIMP protein showed a strong pattern of decline in diestrus, with gelatinase staining significantly lower in DI, followed by another significant decline in DII, and extent of protein detection for all remaining MMPs and TIMPs also decreased by DII (Figure 5). When intensity was scored separately by follicle/structure type, the declines were predominantly observed in DII as compared to other groups (Table 2). Late CLs and atretic follicles were prominent structures in DII in the current study (Table 1). Although MMPs can be upregulated for both CL formation and regression across a variety of species [16,22,24], CL immunostaining intensity in Siberian hamsters declined in the late CLs of DII as compared to early and mid CLs more typically observed in E and DI (Table 2). The current study compared MMP levels from all phases of the natural estrous cycle and did not include a specific luteal time course; however significant changes over the course of the hamster luteal lifespan may well occur and should be examined to better characterize the role of MMPs/TIMPs in the CL.

Examining the changes of MMP immunoreactivity across the cycle highlights the potential influence of gonadal steroid interaction and MMP regulation. Indeed, the observed MMP and TIMP declines during diestrus are concomitant with changes in estradiol concentrations, which peak in proestrus and decline significantly by the diestrus stages (Figure 1). Both the number of estrogenic antral follicles and the intensity of MMP-9 and -14 immunostaining in these follicles declined in DII as compared to all other stages (Tables 1 and 2). Similarly, TIMP-1 immunoreactivity was lower in antral follicles observed in both DI and DII. Estradiol has been shown to regulate MMP expression in several tissues, including mouse uterus [39], and human granulosa cells [40], and given the cyclic nature of MMP expression observed in the current study, may also be influential in Siberian hamster ovaries. Examining MMPs/TIMPs in a late proestrus group would more precisely assess the role of proteases in the Siberian hamster periovulatory period.

Localization for all MMPs and TIMPs showed abundant granulosa and theca cell staining in P and E, and is similar to MMP-2 staining observed in rat preovulatory and antral follicles after PMSG administration [10]. Although staining in adjacent CT-based stroma was low, MMP-2, MMP-9 and TIMP-1 immunoreactivity was noted in the basement membrane of follicles in the present study, and TIMP-1 has been observed in the basement membrane of follicles in PMSG treated rats [10]. Overall, the highest protein levels were observed in the follicle structures and estrous groups associated with growth-follicular growth in P and E, and CL formation in DI, further evidence for MMP and TIMP roles in cell proliferation.

Ovaries of most temperate mammals undergo seasonal regression where healthy ovaries shut down function and maintain in a quiescent state for a period of time before undergoing recrudescence. MMPs -2, -9, -14 and TIMPs -1 and -2 mRNA showed no significant differences during photoperiod mediated ovarian regression compared to LD controls. Ovaries in the regression experiment were collected prior to estrous cycle analysis, and all (including LD controls) were collected at DII, the stage most closely related to regression [5]. This experimental design may mask actual changes in MMPs during regression; however, the ovaries enter a DII-like state as quiescence is initiated, and collecting regressed non-functional ovaries in P or E stage is not a possibility by definition. While regressed stroma and terminal atretic follicles did show some increased in immunostaining intensity (Table 4), overall MMP/TIMP intensity was low throughout the regression ovaries. The general lack of change in MMP mRNA and protein during ovarian regression may suggest that MMPs and TIMPs are present during regression in levels that may be adequate enough to carry out the necessary ovarian remodeling observed, and/or that other processes or mechanisms are involved in the regression process. Gonadal apoptosis is a critical component in photoperiod-mediated regression and peaks following three weeks of SD exposure in Siberian hamster ovaries [5]. A mix of cell death along with low-level MMP and non-MMP protease action may regulate the ovarian atrophy that occurs over 3-14 weeks of SD exposure. This lengthy time interval related to the amount of ovarian remodeling may also explain the general lack of significant increases in protease presence. Ovarian regression is a slow process, taking weeks for the ovary to become regressed and significantly different from the LD ovary, while in the estrous cycle, especially in P, E, and DI, rapid remodeling occurs in a matter of hours and days. Examining action of additional proteases, including additional MMPs/TIMPS, and using a finer scale for the regression timeline will clarify the mechanism of photoperiod-mediated ovarian atrophy, and will aid in the understanding of how non-cycling ovaries are maintained during quiescent periods.

## Conclusions

This study is the first to examine MMPs and TIMPs in both the normal ovarian cycle and during photoperiod-mediated ovarian regression. MMP/TIMP protein staining was high in steroidogenic follicles and large, presumably functional CL, and was generally low in DII as compared to other stages of the estrous cycle. Our results suggest that while MMPs appear to be involved in the normal ovarian estrous cycle at the protein level in hamsters, the MMPs examined (MMP-2, -9, -14) are unlikely to be key players in the slow atrophy of tissue as observed in Siberian hamster ovarian regression.

## Competing interests

The authors declare that they have no competing interests.

## Authors' contributions

LAV performed all experiments and drafted the manuscript. KAY conceived the experimental design, coordinated the performance of the experiments, re-scored all ovarian sections following reviewer's comments (with confirmation by LAV) and contributed to the drafting/revisions of the manuscript. All authors read and approved the final manuscript.
